# Stressed systems: Stroke unit bed occupancy and impact on reperfusion therapy in acute ischemic stroke

**DOI:** 10.3389/fneur.2023.1147564

**Published:** 2023-03-30

**Authors:** Rolf A. Blauenfeldt, Dorte Damgaard, Claus Z. Simonsen

**Affiliations:** ^1^Department of Neurology, Aarhus University Hospital, Aarhus, Denmark; ^2^Department of Clinical Medicine, Aarhus University, Aarhus, Denmark

**Keywords:** reperfusion therapy, ischemic stroke (IS), bed occupancy, quality in care, systems of care

## Abstract

**Objectives:**

We observed a decrease in the number of patients who were offered reperfusion therapy. We aimed to investigate if whether hospital system pressure measured as the percentage of stroke bed occupancy influenced decisions on treatment and disposition.

**Design:**

Data from a regional quality of stroke care database were obtained and linked to the organizational data monitoring of the hourly inpatient stroke bed occupancy rate. Logistic regression was used to analyze the relationship.

**Results:**

A total of 15,025 admissions were included from 1 January 2019 to 24 August 2022. Of these, 5,659 (38%) had an acute ischemic stroke. The rates of reperfusion therapy treatment were the highest in 2019 (36.2%) and 2020 (34.1%) and declined afterward (30.0% in 2021). In the logistic regression analysis, an occupancy rate of ≥85% in the hour of the first admission was associated with reduced odds of admission at the stroke unit within 3 h from the symptom onset [adjusted odds ratio: 0.80, 95% confidence interval: (0.71–0.90), *p* < 0.001] and a reduced odds of receiving reperfusion therapy (adjusted odds ratio: 0.83 (0.73–0.95), *p* = 0.007).

**Conclusion:**

An increased bed occupancy level in the hour of the first hospital admission for stroke patients was strongly associated with decreased odds of receiving reperfusion therapy.

## Background

Recent years have seen immense progress in the treatment of acute ischemic stroke (AIS). Reperfusion therapy using intravenous thrombolysis (IVT) and/or endovascular therapy (EVT) is evidence-based, and indications for their use are constantly expanding (1, 2). The overarching aim is to provide fast and safe reperfusion therapy for all eligible patients. Care in a specialized stroke unit is further associated with improvements in patient outcomes (3). A high bed occupancy rate (BOR) has been associated with increased admission threshold in emergency departments, and in some studies, it has also been associated with increased mortality (4–7). At the stroke unit, a high BOR might be associated with reduced quality of stroke care and a lower likelihood of direct admission to the stroke unit (8). It is unknown whether BOR may have an impact on the proportion of AIS patients who were offered stroke unit admission and reperfusion therapy.

After an organizational change resulting in a longer delay in transferring patients for rehabilitation, we saw an increased BOR at our acute stroke unit. Although the number of admissions was constant, the number of acutely admitted patients declined, and we saw a decline in reperfusion therapy rates as well. In this study, we aim to investigate if the increased BOR had an impact on whether patients with symptoms of AIS were offered acute admission and treatment at the stroke center.

## Methods

The Department of Neurology at Aarhus University Hospital serves a catchment area of 900,000 patients for IVT and 1.3 million for EVT, and at night and weekends, this area is expanded to 1.9 million people for EVT. Patients with suspected AIS are initially assessed by the emergency medical service in the prehospital field, and a teleconference with the on-call neurologist is initiated. Based on the history and the clinical findings, a decision regarding admission to the stroke unit is made. If the history is clear and a stroke is suspected, the patient is admitted directly to the stroke center. If a stroke is deemed unlikely, the patient is admitted to the nearest emergency department for a workup. Prehospital triage is a standard procedure in Denmark and has been described elsewhere (9). The teleconference was conducted by a stroke neurologist or a resident, who could bring on the stroke neurologist to the call. Live bed occupancy rates at the stroke ward are always visible using an electronic patient flow management system displayed in the call room and on each computer.

The stroke ward has 19 beds and operates independently from the neuro-intensive care unit.

It is mandated by the government to measure the quality of care for stroke and transient ischemic attack. This is collected and stored in the Database of the Danish Stroke Center (DDSC). Organizational data are monitored in a regional Business Intelligence database, and the number of occupied beds is monitored on an hourly basis to optimize the use of resources.

On 4 May 2020, the centralization of rehabilitation beds was performed, so rehabilitation patients then could not be discharged to outside hospitals. This resulted in more patients waiting for rehabilitation in stroke unit beds and an increased BOR at the stroke unit.

We hypothesized that if the stroke unit is full (high BOR), this would influence the odds of receiving reperfusion therapy treatment.

We extracted data from the DDSC and the Business Intelligence database in the period (1 January 2019–24 August 2022). We linked all admissions in the period with those as follows:

1. Average stroke unit BOR on the day of the first admission.
2. Stroke unit BOR in the hour of the first admission.
3. Stroke unit BOR 3 h before the time of the first admission.

The time of first admission was defined as the time of arrival at the first hospital door (either directly to the stroke unit for patients with suspected stroke or to the emergency department at local hospitals for patients, that were deemed unlikely to have a stroke). A full stroke unit was defined as having a BOR of ≥85%. This level is generally accepted to reflect exhaustion of the buffer capacity (10).

Since the study is performed as part of a quality assurance project, it is exempted from ethical approval.

It was not appropriate or possible to involve patients or the public in the design, conduct, report, or dissemination plans of our research.

**Figure 1:**
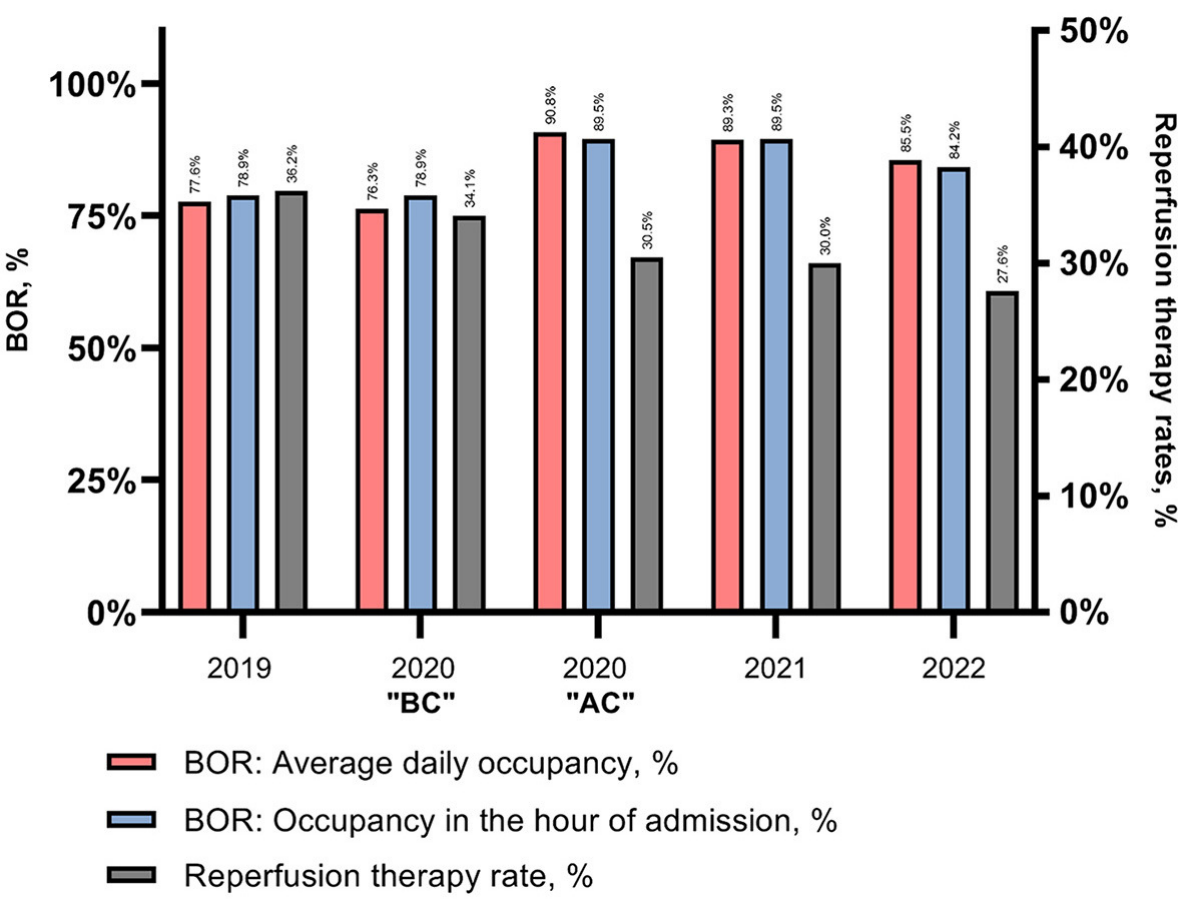
Stroke center BOR and reperfusion therapy rates. Bed occupancy from 2019-2022 examined on the day of admission and in the hour of admission. BOR, Bed occupancy rate; BC, Before centralization of rehabilitation beds; AC, After centralization.

Baseline characteristics were presented in numbers and percentages or median and interquartile range (IQR) as appropriate. Tests of differences were performed using the ANOVA for normally distributed data and the non-parametric Wilcoxon rank-sum (two groups) or the Kruskal–Wallis test (>2 groups) for non-normally distributed data. Logistic regression analyses (uni- and multivariable) were used to examine predictors for receiving reperfusion therapy (intravenous thrombolysis and/or mechanical thrombectomy). Variables that were deemed clinically relevant for the outcome measure were included in the model, and they were BOR (continuous and dichotomized at </≥ 85%), known symptom onset (yes/no), independent in daily activities of living (modified Rankin Scale score of 0–2; yes/no), baseline National Institute Of Health Stroke Scale (NIHSS, ranging from 0 to 42), known or newly diagnosed diabetes (yes/no), and known or newly diagnosed atrial fibrillation (yes/no). Reperfusion therapy rates were defined as the proportion of AIS patients receiving reperfusion therapy out of all AIS patients admitted to the stroke unit. The final diagnosis was based on the discharge diagnosis as reported to the Danish Stroke Registry. For all tests, statistical significance was defined as *p*-values of <0.05. Analysis was performed as a complete-case analysis. Stata 15.1 software (StataCorp, College Station, Texas, USA) was used for data management and statistics and GraphPad Prism 9.5.0 (GraphPad Software, San Diego, California, USA) is used for Figure 1.

## Results

A total of 15,025 admissions were included, 5,659 with AIS, 899 with intracerebral hemorrhage, and 2,025 with TIA. Non-stroke diagnoses accounted for 5,720 admissions. The remaining 722 admissions were other cerebrovascular diseases, where amaurosis fugax/central retinal artery occlusion accounted for 388. In patients with AIS, 41.4% were women, and the median (IQR) age was 73 (64, 81) years. There were no significant differences by the year of admission in age, sex, or comorbidities in the study period (see Table 1), and the number of AIS admissions was constant [AIS admissions per day (mean) was 4.1 in 2019, 4.3 to 4.4 in 2020, 4.4 in 2021, and 4.1 in 2022]. The distribution of all (stroke and non-stroke) daily stroke center admissions was also constant. These admissions are visualized in Supplementary Figure 1.

**Table 1:** Characteristics of AIS admissions during the study period.

**Notes**	**Total**	**2019**	**2020**	**2020**	**2021**	**2022**	***p*-value**
			**Before centralization (BC)**	**After centralization (AR)**			
*n*	5,659	1,491	533	1,069	1,610	956	
Age, median (IQR)	73 (64, 81)	73 (63, 81)	73 (64, 81)	73 (63, 81)	74 (64, 81)	74 (65, 81)	0.12
Female, *n* (%)	2,342 (41.4%)	621 (41.5%)	233 (41.5%)	430 (40.2%)	678 (42.1%)	392 (41.0%)	0.90
Diabetes, *n* (%)	954 (16.9%)	241 (16.2%)	85 (15.9%)	195 (18.2%)	273 (17.0%)	160 (16.8%)	0.68
Atrial fibrillation, *n* (%)	1,142 (20.2%)	318 (21.3%)	104 (19.5%)	202 (18.9%)	331 (20.6%)	187 (19.6%)	0.59
Known onset, *n* (%)	2,765 (48.9%)	733 (49.2%)	260 (48.8%)	523 (48.9%)	786 (48.8%)	489 (48.6%)	1.00
NIHSS, median (IQR)	3 (1, 7)	4 (1, 8)	4 (2, 8)	3 (1, 7)	3 (2, 8)	3 (1, 7)	0.10
Onset to stroke unit admission (duration), median minutes (IQR)	299 (95, 1,007)	249 (93, 927)	302 (99, 885)	349 (104, 1,145)	316 (95, 1,000)	289 (90, 1,060)	0.16
IVT, *n* (%)	1,372 (24.2%)	417 (28.0%)	147 (27.6%)	248 (23.2%)	353 (21.98%)	207 (21.7%)	< 0.001
EVT, *n* (%)	771 (13.6%)	232 (15.6%)	72 (13.5%)	138 (12.9%)	225 (14.0%)	104 (10.9%)	0.02
Reperfusion therapy (IVT and/or EVT), *n* (%)	1,794 (31.7%)	539 (36.2%)	182 (34.1%)	326 (30.5%)	483 (30.0%)	264 (27.6%)	< 0.001
First admitted to emergency room, *n* (%)	1,006 (18.2%)	252 (16.9%)	87 (16.3%)	184 (17.2%)	317 (19.7%)	199 (20.8)	0.04
Acute admission (candidates for reperfusion therapy), *n* (%)	2,568 (45.4%)	730 (49%)	255 (47.8%)	471 (44.1%)	705 (43.8%)	407 (42.8%)	0.008
Length of stay at stroke unit, median (IQR)	2 (1, 5)	2 (1,3)	2(1,3)	2 (1,5)	3 (1,7)	2 (1,7)	< 0.001

Data describing age, sex, comorbidities, and stroke severity in AIS patients.

AIS, acute ischemic stroke; EVT, endovascular therapy; IVT, intravenous thrombolysis; NIHSS, National Institute of Health Stroke Scale.

Separating admissions into acute admission (patients admitted as candidates for reperfusion therapy) and non-acute admissions, this rate declined from 49% in 2019 to 43.8% in 2021, *p* = 0.004. We saw a median increase in the length of stay from a median (IQR) of 2 (1, 3) days in 2019 to 3 (1, 7) days in 2021, *p* < 0.001.

The rates of reperfusion therapy treatment in patients with AIS were the highest in 2019 (34.1%) and 2020 (36.2%) and were followed by a significant decline in 2020 and onwards (30.0% in 2021), see Table 1.

Following the centralization of rehabilitation beds in 2020, the daily BOR increased from 76.3 to 90.8% on average.

As already mentioned, we examined the BOR not only as an average occupancy during the day of admission but also in the hour of the first hospital admission. Figure 1 illustrates this over the study period.

In the univariable logistic regression analysis, average daily BOR (per percent increase) was negatively associated with odds for AIS patients receiving reperfusion therapy [adjusted OR 0.995 (0.991–0.999), *p* = 0.019]. No significant impact of daily average BOR of 85% or above on odds for reperfusion therapy was seen (unadjusted and adjusted). However, the analysis using the hourly BOR at the hour of admission was significantly associated with reduced odds of receiving reperfusion therapy (adjusted and unadjusted). A BOR of at least 85% in the hour of the first admission was associated with reduced odds [OR 0.83 (0.73–0.95)] of receiving reperfusion therapy (*p* = 0.007) in the adjusted model (see Table 2).

**Table 2:** Association between bed occupancy and odds for reperfusion therapy and admission within 3 h from the symptom onset.

**Odds for reperfusion therapy**	**Odds ratio (univariable)**	**95% CI**	***p*-value**	Adj Odds ratio^*^	**95% CI**	***p*-value**
**Average daily occupancy**						
Average daily occupancy rate, median percent (IQR)	0.995	0.991–0.999	0.019	0.996	0.992–1.001	0.100
Average daily occupancy rate ≥85%, *n* (%)	0.901	0.805–1.007	0.067	0.916	0.803–1.05	0.195
**Hourly occupancy**						
Occupancy rate in the hour of first admission, median percent (IQR)	0.992	0.989–0.995	< 0.001	0.993	0.990–0.997	0.001
Occupancy rate at time of first admission ≥85%, *n* (%)	0.811	0.723–0.908	< 0.001	0.83	0.73–0.952	0.007
**Odds for admission within 3 h from onset**	**Odds ratio (univariable)**	**95% CI**	***p*** **-value**	**Adj Odds ratio** ^**^	**95% CI**	***p*** **-value**
Occupancy rate in the hour of first admission, median percent (IQR)	0.993	0.990–0.996	< 0.001	0.993	0.990–0.997	< 0.001
Occupancy rate at time of first admission ≥85%, *n* (%)	0.785	0.704–0.877	< 0.001	0.802	0.713–0.902	< 0.001

* Adjusted for known symptom onset (yes/no), independent in daily activities of living (modified Rankin Scale score < 3; yes/no), baseline NIHSS, known or newly diagnosed diabetes (yes/no), and known or newly diagnosed atrial fibrillation (yes/no).

** Adjusted for independence in daily activities of living (modified Rankin Scale score < 3; yes/no), baseline NIHSS, known or newly diagnosed diabetes (yes/no), and known or newly diagnosed atrial fibrillation (yes/no).

The same results were found for BOR 3 h before the first admission (data not shown). Analyses were repeated with both IVT and EVT as dependent variables, and no meaningful difference in the estimates was found (data not shown).

**Figure 2:**
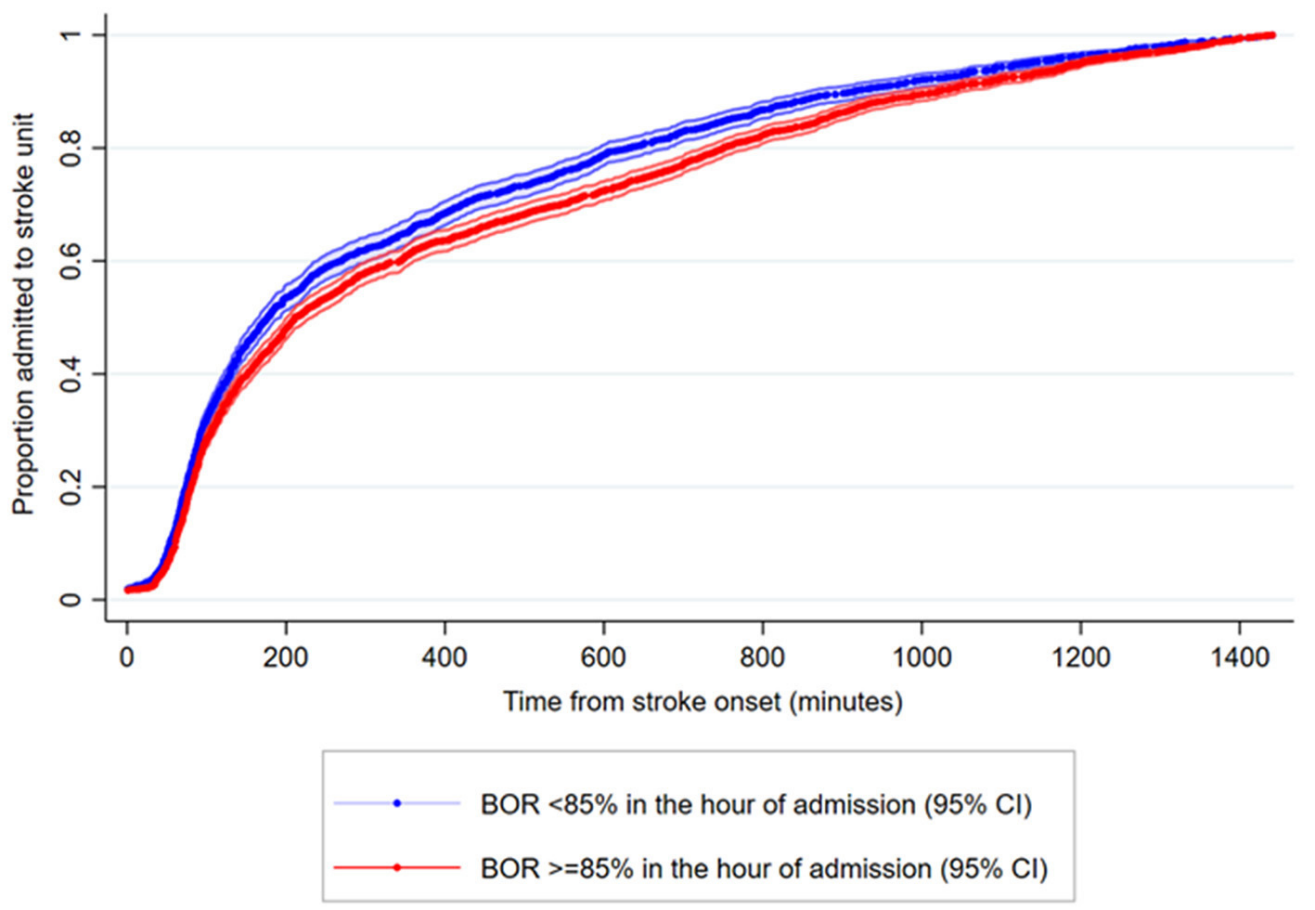
Proposition of ischemic stroke patients admitted to a stroke unit in relation to time from onset. Cumulative data on time from stroke onset to stroke unit admission in AIS patients stratified by bed occupancy rate ≥ 85% (red curve) or BOR < 85% (blue curve) in the hour of admission. When bed occupancy rate was high, some patients were not admitted directly to the stroke unit. They were instead admitted and diagnosed at an emergency department, and afterwards transferred to the stroke unit often outside the reperfusion treatment window. The two curves merge, illustrating that the stroke patients eventually end at the stroke unit. In 134 out of 5,659 (2.4%) AIS admissions no valid time registrations were available and these were omitted. Only admissions with an onset to admission duration of ≤24 hours are visualized (4,379 out of 5,525 [79.3%]).

The effect of BOR on the proportion of patients admitted in relation to onset-to-admission duration is visualized in Figure 2.

When BOR is >85% (red curve in Figure 2), fewer patients are admitted acutely but referred to the nearest emergency department at a local hospital. In case of a stroke diagnosis, they are then transferred to the stroke unit later, but often outside the treatment window for reperfusion therapy. In a logistic regression analysis, a BOR at the hour of admission of 85% or above was associated with decreased odds of being admitted to the stroke unit within 3 h from the symptom onset [OR 0.80 (0.71–0.90), *p* < 0.001; adjusted and unadjusted].

In patients who were treated with reperfusion therapy, there were no changes in the proportion of patients with mild strokes (NIHSS 0–4), distribution of different epochs of onset-to-arrival time, or imaging modality (Table 3).

**Table 3:** Onset to admission time in patients treated with reperfusion therapy treatment stratified by year.

	**2019**	**2020**	**2020**	**2021**	**2022**	***p*-value**
		**Before centralization (BC)**	**After centralization (AR)**			
	***n** = **561***	***n** = **188***	***n** = **414***	***n** = **693***	***n** = **369***	
Admitted within the 4.5 h time window, *n* (%)	176 (31.4%)	54 (28.7%)	118 (28.5%)	193 (27.8%)	115 (31.2%)	0.63
Admitted in 4.5–6 h time window, *n* (%)	23 (4.1%)	7 (3.7%)	10 (2.4%)	22 (3.2%)	10 (2.7%)	0.61
Admitted in 6–24 h time window, *n* (%)	87 (15.5%)	35 (18.6%)	75 (18.1%)	128 (18.5%)	78 (21.1%)	0.29
Unknown onset (last known well), *n* (%)	153 (40.5%)	50 (38.8%)	109 (39.4%)	166 (35.2%)	101 (37.3%)	0.58

The majority of patients first seen at the emergency department were independent in activities of daily living before their stroke. In 2019, 84.5% of patients first seen at the emergency department had a prestroke mRS of ≤2, whereas this was 75.7% in 2021 (*p* < 0.001). The remaining patient characteristics did not change significantly (Table 4).

**Table 4:** Characteristics of patients first admitted to the emergency room stratified by year.

**Characteristics of patients first admitted to the emergency room**	**2019**	**2020**	**2020**	**2021**	**2022**	***p*-value**
		***Before rehab***	***Post rehab***			
	***n** = **252***	***n** = **87***	***n** = **184***	***n** = **317***	***n** = **199***	
Female, *n* (%)	104 (41.3%)	39 (44.8%)	86 (46.7%)	155 (48.9%)	87 (43.7%)	0.45
Age, median (IQR)	75 (64.5, 81)	74 (62, 82)	74 (63, 81)	75 (64, 83)	76 (69, 82)	0.37
Diabetes, *n* (%)	62 (24.6%)	19 (21.8%)	33 (17.9%)	82 (26.0%)	47 (23.6%)	0.33
Atrial fibrillation, *n* (%)	52 (20.6%)	15 (17.2%)	33 (17.9%)	87 (27.6%)	43 (21.6%)	0.063
Living in nursing home, *n* (%)	17 (6.8%)	11 (12.6%)	17 (9.4%)	30 (9.7%)	16 (8.3%)	0.52
Prestroke modified Rankin Scale ≤ 2, median (IQR)	207 (84.5%)	65 (74.7%)	137 (75.7%)	231 (75.7%)	150 (83.3%)	0.031
Onset to admission (first door) within 4.5 h, *n* (%)	96 (38.1%)	32 (36.8%)	69 (37.5%)	114 (36.0%)	76 (38.2%)	0.98
NIHSS, median (IQR)	3 (1, 7)	3.5 (1, 7)	2 (1, 5)	3 (1, 8)	3 (1, 6)	0.22
Reperfusion therapy, *n* (%)	36 (14.3%)	8 (9.2%)	15 (8.2%)	28 (8.8%)	17 (8.5%)	0.14

Data describing age, sex, comorbidities, and stroke severity in AIS patients.

NIHSS, National Institute of Health Stroke Scale.

Similarly, the distribution of neurological deficits on the National Institute of Health Stroke Scale (NIHSS) did not change over time in patients first admitted to the emergency department (Supplementary Table 1).

## Discussion

We found that the chance of receiving reperfusion therapy was reduced by 17% if the BOR at the stroke unit was more than 85% at the hour of admission. The chance of receiving reperfusion therapy was also significantly lower when the occupancy level was seen as a per percent increase in the daily average occupancy. For a 1% increase in BOR, the treatment rate fell by 0.7%. The hourly BOR before and at the time of admission was strongly associated with both decreased chances of admission within 3 h from the symptom onset and decreased chance of receiving reperfusion therapy. This indicates that fewer patients were accepted as candidates for reperfusion therapy when the occupancy level was high. There were no changes in age, comorbidities, or stroke severity during the observational period, so these factors could not explain the change in the treatment rate. The increased BOR was not a result of increased admissions or due to patients having more comorbidities, but a consequence of a slower discharge to rehabilitation. Our data suggest that a busy stroke unit quantified/measured as a high BOR could be a boundary to effective treatment and quality care.

Centralization and using hospital resources to a maximum is a common scenario. The influence on care is largely unknown. In intensive care medicine, awareness of the scarcity of resources such as available beds is well described (“ritual of last bed”) and has been found to affect the admission and discharge decisions of physicians (11, 12). In a previous multicenter register study from Sweden, stroke unit BOR was negatively associated with direct stroke center admission from the emergency department (8). However, heterogeneity between hospitals was found, and some hospitals had buffer systems in place so that stroke unit care could be preserved despite increased pressure. It may, together with the current study, suggest that stroke physician behavior and decisions regarding admission may be influenced by stroke bed availability. Cognitive biases are well described and affect the clinical decision-making process (13, 14). In addition, cognitive depletion, such as fatigue, mood changes, or stress, may predispose physicians toward implicit decision-making that requires the least mental energy (13). Marginal cases with an atypical presentation, or premorbid non-independent patients, may make stroke physicians direct patients to an emergency department instead of a stroke unit. The COVID-19 pandemic did affect stroke care in some countries, but based on earlier performed analysis indicating no effect on admission, reperfusion therapy rates, and quality of acute stroke care, it is unlikely that the pandemic affected the results of the current study (15, 16).

The current study is limited by its retrospective and single-center study design. The use of an 85% bed occupancy rate as cut off is likely an oversimplification and may not reflect stress on the system or risk of access or buffer capacity block (10, 17). To account for this, we also analyzed BOR as a continuous measure and found similar results as in the dichotomized model. The main strength of this study is the ability to link mandatory stroke registry data with detailed organizational data on bed occupancy at a level of granularity that provides hourly data. We believe the robustness of the findings is supported by the coherence between unadjusted and adjusted results.

It was not within the scope of this study to identify solutions to the increased BOR. Before the current study period, a reorganization of the stroke/TIA care was performed with the establishment of a specialized outpatient clinic, reducing the stroke ward admissions and bed-day use (18). Stroke systems of care are complex, and increasing the number of stroke “buffer” beds is not a likely long-lasting solution. The outflow of patients to rehabilitation facilities and the rehabilitation in the municipalities needs to be assessed as well. Computer modeling of the entire system of stroke care using stochastic methods that capture the variation in arrivals, lengths of stay, and discharge delays may be a possible future solution (19). Future demands for stroke unit beds should be included as well (20). The current study has resulted in the opening of two additional stroke “buffer” beds while awaiting a more definite solution.

## Conclusion

We found a decreased rate of reperfusion therapy treatment following a rise in the bed occupancy level. Increased BOR at the hour of first hospital admission for stroke patients was strongly associated with decreased odds of receiving reperfusion therapy. Quality in treatment seemed affected by how busy the stroke unit was.

## Strengths and limitations of this study

- This is the first study to directly investigate whether increased bed occupancy rate has an impact on whether patients with symptoms of acute ischemic stroke were offered acute admission and treatment at the stroke center.
- We included all acute ischemic stroke admissions in period 1 January 2019 to 24 August 2022.
- The main strength of this study is the ability to link mandatory stroke registry data with detailed organizational data on bed occupancy at a level of granularity that provides hourly data.
- The robustness of the findings is supported by the coherence between unadjusted and adjusted models and bed occupancy level as a dichotomized and continuous measure.
- The study is limited by its retrospective and single-center study design.

## Data availability statement

The datasets presented in this article are not readily available because of ethical and privacy restrictions. Requests to access the datasets should be directed at: RB, rolfblau@rm.dk.

## Author contributions

RB, DD, and CS researched the literature and conceived the study. RB performed the data analysis and wrote the first draft of the manuscript. All authors were involved in the critical interpretation of data, reviewed and edited the manuscript, and approved the final version of the manuscript.
