# Supplementary material for: Stressed systems: Stroke unit bed occupancy and impact on reperfusion therapy in acute ischemic stroke
Source: Front Neurol. 2023 Mar 30;14:1147564. doi: 10.3389/fneur.2023.1147564 (PMC10100068; doi:10.3389/fneur.2023.1147564)
Supplement: Supplementary file 1 [file Data_Sheet_1.PDF]

Supplemental Figure 1 – Distribution of daily admissions during the study period

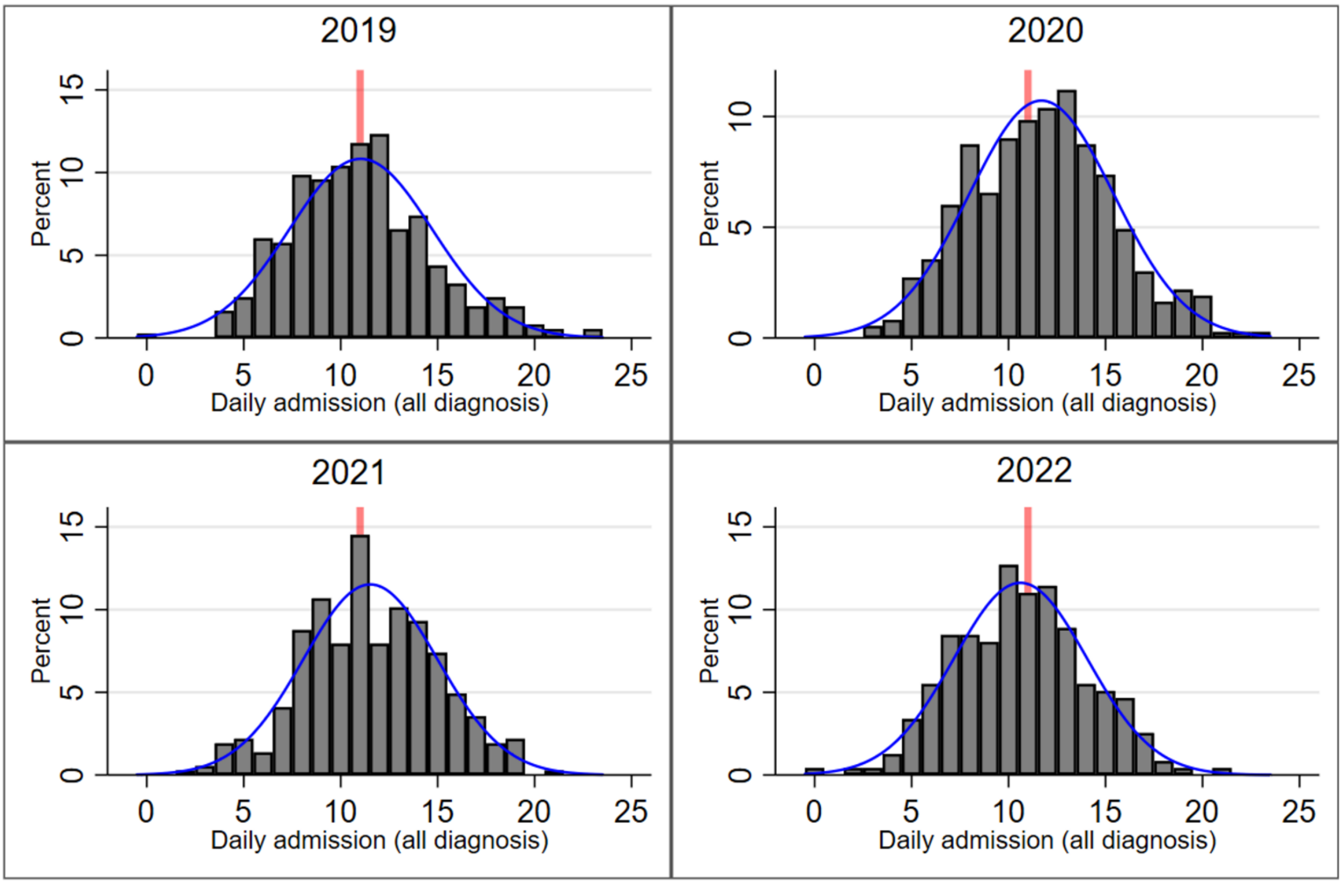

Overall median (IQR): 11 (9, 14) admissions per day, **2019:** median (IQR): 11 (8, 13), **2020:** median (IQR): 12 (9, 14), **2021:** median (IQR): 11 (9, 14) and **2022:** median (IQR): 11 (8, 13).

Legends: Daily admissions (stroke and non-stroke diagnosis) at the stroke unit and visits to the acute stroke/TIA clinic during the study period. Red line in each histogram marks the overall median daily admission number during the entire study period. The blueline represents a normal-density plot.

Supplemental material – Table 1:

Neurological deficits recorded at stroke center arrival in patients first admitted to the emergency room stratified by year.

**Neurological deficits in patients first admitted to an emergency room**	**2019**	**2020**	**2020**	**2021**	**2022**	**p-value**
		Before rehab	*Post rehab*			
	n=252	*n=87*	*n=184*	*n=317*	*n=199*	
1a. Level of Consciousness (affected yes/no), n(%)	20 (7.9%)	5 (5.7%)	10 (5.4%)	32 (10.1%)	12 (6.0%)	0.27

1b. LOC Questions (affected yes/no), n(%)	67 (26.6%)	27 (31.0%)	41 (22.3%)	88 (27.8%)	49 (24.6%)	0.53
1c. LOC Commands (affected yes/no), n(%)	33 (13.1%)	12 (13.8%)	26 (14.1%)	43 (13.6%)	19 (9.5%)	0.66
2. Best Gaze (affected yes/no), n(%)	27 (10.7%)	11 (12.6%)	20 (10.9%)	48 (15.1%)	17 (8.5%)	0.21
3. Visual (affected yes/no), n(%)	48 (19.0%)	14 (16.1%)	39 (21.2%)	59 (18.6%)	39 (19.6%)	0.89
4. Facial Palsy (affected yes/no), n(%)	78 (31.0%)	29 (33.3%)	63 (34.2%)	121 (38.2%)	70 (35.2%)	0.50
5a. Motor Arm (right) (affected yes/no), n(%)	60 (23.8%)	18 (20.7%)	26 (14.1%)	65 (20.5%)	30 (15.1%)	0.057
5b. Motor Arm (left) (affected yes/no), n(%)	49 (19.4%)	21 (24.1%)	34 (18.5%)	70 (22.1%)	35 (17.6%)	0.59
6a. Motor Leg (right) (affected yes/no), n(%)	55 (21.8%)	19 (21.8%)	27 (14.7%)	64 (20.2%)	33 (16.6%)	0.28
6b. Motor Leg (leftt) (affected yes/no), n(%)	60 (23.8%)	17 (19.5%)	33 (17.9%)	64 (20.2%)	46 (23.1%)	0.56
7. Limb Ataxia (affected yes/no), n(%)	57 (22.6%)	15 (17.2%)	43 (23.4%)	51 (16.1%)	35 (17.6%)	0.17
8. Sensory (affected yes/no), n(%)	60 (23.8%)	16 (18.4%)	39 (21.2%)	77 (24.3%)	32 (16.1%)	0.18
9. Best Language (affected yes/no), n(%)	64 (25.4%)	26 (29.9%)	36 (19.6%)	78 (24.6%)	47 (23.6%)	0.41
10. Dysarthria (affected yes/no), n(%)	81 (32.1%)	31 (35.6%)	45 (24.5%)	109 (34.4%)	63 (31.7%)	0.19
11. Extinction and Inattention (formerly Neglect) (affected yes/no), n(%)	19 (7.5%)	7 (8.0%)	10 (5.4%)	29 (9.1%)	24 (12.1%)	0.21

LOC: Level of consciousness
